# Non-Target Activity Detection by Post-Radioembolization Yttrium-90 PET/CT: Image Assessment Technique and Case Examples

**DOI:** 10.3389/fonc.2014.00011

**Published:** 2014-02-03

**Authors:** Yung Hsiang Kao, Andrew E. H. Tan, Richard H. G. Lo, Kiang Hiong Tay, Bien Soo Tan, Pierce K. H. Chow, David C. E. Ng, Anthony S. W. Goh

**Affiliations:** ^1^Department of Nuclear Medicine and PET, Singapore General Hospital, Singapore; ^2^Department of Nuclear Medicine, Austin Hospital, Melbourne, VIC, Australia; ^3^Department of Diagnostic Radiology, Singapore General Hospital, Singapore; ^4^Department of General Surgery, Singapore General Hospital, Singapore; ^5^Department of Surgical Oncology, National Cancer Centre Singapore, Singapore; ^6^Office of Clinical Sciences, Duke-NUS Graduate Medical School, Singapore

**Keywords:** yttrium-90 PET/CT, bremsstrahlung SPECT/CT, non-target activity, yttrium-90 radioembolization, selective internal radiation therapy, diagnostic reporting technique, yttrium-90 resin microspheres, SIR-spheres

## Abstract

High resolution yttrium-90 (^90^Y) imaging of post-radioembolization microsphere biodistribution may be achieved by conventional positron emission tomography with integrated computed tomography (PET/CT) scanners that have time-of-flight capability. However, reconstructed ^90^Y PET/CT images have high background noise, making non-target activity detection technically challenging. This educational article describes our image assessment technique for non-target activity detection by ^90^Y PET/CT, which qualitatively overcomes the problem of background noise. We present selected case examples of non-target activity in untargeted liver, stomach, gallbladder, chest wall, and kidney, supported by angiography and ^90^Y bremsstrahlung single-photon emission computed tomography with integrated computed tomography (SPECT/CT) or technetium-99m macroaggregated albumin SPECT/CT.

## Introduction

Radioembolization (RE) is brachytherapy by arterially injected yttrium-90 (^90^Y) microspheres for the treatment of malignancies. Coincidence imaging of ^90^Y is possible because of a minor decay branch to the O^+^ first excited state of zirconium-90 leading to low abundance internal pair production (1–3). Today, high resolution ^90^Y imaging of post-RE microsphere biodistribution may be achieved by conventional positron emission tomography with integrated computed tomography (PET/CT) scanners that have time-of-flight capability (1). However, the optimum image acquisition and reconstruction protocols are still the subject of on-going research across a wide range of scanner types.

For qualitative diagnostic reporting of ^90^Y PET/CT, two aspects should always be addressed, i.e., the biodistribution of target and non-target activity. The presence of non-target activity may have clinical implications for radiomicrosphere toxicity and is as important as target activity detection. However, non-target activity detection by ^90^Y PET/CT is technically challenging. Today’s time-of-flight PET/CT scanners use lutetium-based scintillation crystals, which have intrinsic background activity due to naturally occurring lutetium-176. The combination of intrinsic background activity and a very low ^90^Y positron fraction results in high levels of noise in reconstructed ^90^Y PET/CT images, which at the outset, seem uninterpretable.

Recently, we developed an image assessment technique for non-target activity detection by ^90^Y PET/CT, which qualitatively overcomes the problem of background noise. This is an educational article highlighting the basic principles of non-target activity detection by ^90^Y PET/CT. For technical illustration, we have selected six case examples to present, which include the untargeted liver, stomach, gallbladder, chest wall, and kidney.

## ^90^Y PET Imaging Protocol

Our imaging protocols for ^90^Y PET/CT, ^90^Y bremsstrahlung SPECT/CT, and ^99m^Tc MAA SPECT/CT have been described in detail elsewhere (4–6). Briefly, for ^90^Y PET, our scanner is the GE Discovery 690 PET/CT (General Electric Medical Systems, Milwaukee, WI, USA) with cerium-activated lutetium–yttrium–oxyorthosilicate (LYSO) crystals; positron fraction 3.186 × 10^−5^; half-life 64.1 h; 15 min per bed position; one to two bed positions from the diaphragm downwards to cover the entire liver; image reconstruction by three-dimensional ordered subset expectation maximization (3D-OSEM) algorithm incorporating time-of-flight and point spread function information; 3 iterations and 18 subsets (4).

## Image Assessment Technique

To provide the reader with a rational basis for each diagnosis of non-target activity, all presented cases are correlated to angiography and further supported by ^90^Y bremsstrahlung single-photon emission computed tomography with integrated computed tomography (SPECT/CT) or technetium-99m (^99m^Tc) macroaggregated albumin (MAA) SPECT/CT.

Our image assessment technique for non-target activity detection centers on continuity-of-care and a thorough understanding of case-specific angiography, in close collaboration with interventional radiologists. These two components are paramount as they provide the relevant clinical, angiographic, and dosimetric context to the observed ^90^Y biodistribution and focus the operator onto case-specific regions-at-risk (4).

Firstly, the operator should actively adjust the upper PET visual display threshold setting to deliberately *increase* the background noise to moderate levels. This counter-intuitive action is necessary because non-target activity is often of lower visual intensity than noise spikes (4). If the upper PET visual display threshold had remained at the settings used to suppress the background noise for target activity assessment, it will be unlikely for the operator to detect visually subtle, trace non-target activity. The lower PET visual display threshold setting is 0 kBq/ml (4).

Next, the operator should carefully inspect the rotating maximum intensity projection (MIP) image for any activity protruding from the regular outline of targeted tissue in a non-random pattern, amidst background noise. Finally, the PET and PET/CT images are reviewed in trans-axial, coronal, and saggital planes. Non-target activity is characterized by a non-random pattern of activity localizing to an untargeted anatomical structure on CT. A qualitative diagnosis of non-target activity on ^90^Y PET/CT should be based on its *pattern* and whether it *conforms* to underlying anatomy, not by its visual intensity (4). The presence of a plausible vascular etiology will greatly support a ^90^Y PET/CT diagnosis of non-target activity, although this is not strictly essential because a culprit vessel may not always be identified.

It is not essential to consider the presence or absence of correlative clinical signs or symptoms when making a diagnosis of non-target activity because clinical sequelae is a quantitative function of dose–response radiobiology over time, with no bearing on the qualitative presence of non-target activity at the time of scan. Similarly, it may sometimes be difficult to qualitatively distinguish noise spikes from genuine non-target activity. However, such indeterminate foci are usually too mild to result in any clinically relevant toxicity even if genuine, and therefore do not often impact post-RE management.

Parts of extra-hepatic viscera, which are closely adjacent to the liver (e.g., gallbladder fundus, gastric lesser curve, pylorus, proximal duodenum) are often anatomically inseparable from the liver, making non-target activity detection in these areas very challenging. This problem is further compounded by varying degrees of PET/CT mis-registration due to the relatively long ^90^Y PET acquisition time. However, these issues similarly affect ^90^Y bremsstrahlung SPECT/CT and hence should not be viewed as a comparative disadvantage.

Knowledge of the non-target absorbed dose may guide appropriate mitigative action to minimize non-target radiation toxicity. Hence the detection of non-target activity should immediately be followed by an assessment of the risk of developing clinically significant radiation toxicity. This should be based on ^90^Y PET quantification of the non-target absorbed dose, except in cases of visually subtle, trace non-target activity where the absorbed doses are unlikely to be clinically relevant. The topic of non-target absorbed dose quantification by ^90^Y PET and tissue dose–response is discussed elsewhere (5).

## Case Examples

The six case examples presented here were selected from a 23-patient cohort of predominantly hepatocellular carcinoma patients treated with ^90^Y resin microsphere RE, described in detail elsewhere (4). Of these 23 patients, 8 (34.8%) were detected to have non-target activity by ^90^Y PET/CT. Untargeted liver was the most common site of non-target activity (3/8); only one example is presented here for illustrative purposes. The other five cases of non-target activity involve the stomach (2/8), gallbladder (1/8), chest wall (1/8), and kidney (1/8). The non-target findings on ^90^Y PET/CT were conclusive in all cases. There were no cases of undetected clinically significant non-target activity based on a retrospective review of medical records at a median follow-up of 5.4 months (4).

Case 1: untargeted liver (Figure 1)
Case 2: stomach (Figure 2)
Case 3: stomach (Figure 3)
Case 4: gallbladder (Figures 4 and 5)
Case 5: chest wall (Figures 6 and 7)
Case 6: kidney (Figure 8)

**Figure 1:**
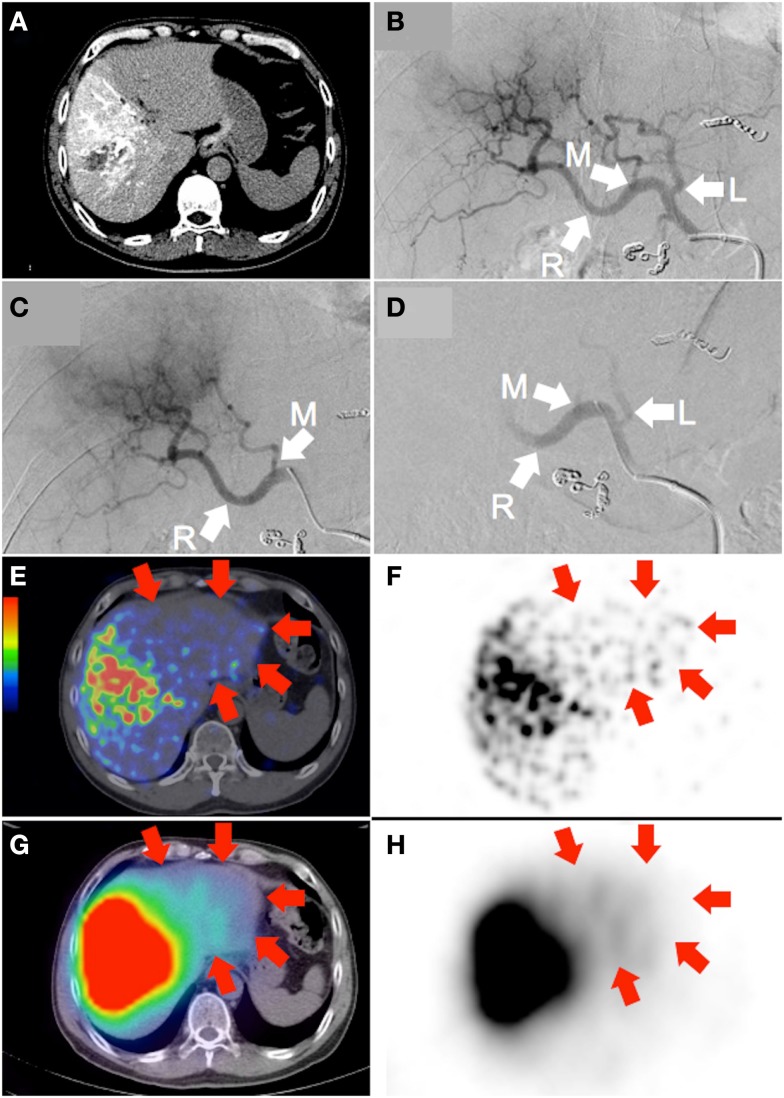
**Large hepatocellular carcinoma of the right lobe (actual tumor not well depicted)**. **(A)** Catheter-directed CT angiogram of the right hepatic artery, proximal to the origin of the middle hepatic artery, delineates the target arterial territory. **(B)** Digital subtraction angiography (DSA) of the proper hepatic artery demonstrates the hepatic arterial tree. The liver is supplied by the left hepatic artery (“L”; branch of the common hepatic artery), middle hepatic artery (“M”; branch of the right hepatic artery) and right hepatic artery (“R”; continuation of the common hepatic artery). Prophylactic coil embolization of the gastroduodenal, right gastric, and accessory left gastric arteries were performed. **(C)** DSA of the target arterial tree with the catheter tip in the right hepatic artery, proximal to the origin of the middle hepatic artery, immediately prior to RE. **(D)** Moderate vascular stasis and contrast reflux into the left and proper hepatic arteries is seen on DSA immediately after RE, with no change in catheter tip position. **(E,F)** ^90^Y PET/CT depicts in high resolution, non-target activity in a non-random distribution conforming to the anatomy of the untargeted left liver lobe (arrows). **(G,H)** ^90^Y bremsstrahlung SPECT/CT shows concordant but subtle diffuse non-target activity in the untargeted left liver lobe (arrows).

**Figure 2:**
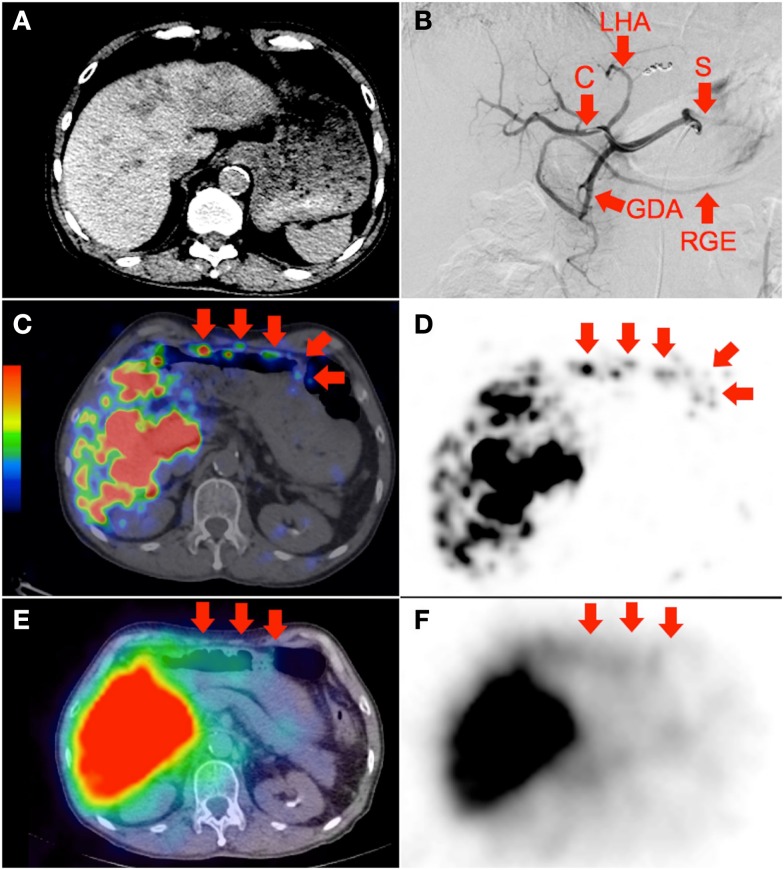
**(A)** Multifocal hepatocellular carcinoma with predominantly left lobe disease seen on non-contrast-enhanced CT. The patient was recently treated with sorafenib, a multi-kinase inhibitor with anti-angiogenic properties. RE was performed in two injections via the left and right hepatic arteries. Prophylactic coil embolization of the gastroduodenal artery was not performed at the discretion of the interventional radiologist. **(B)** Digital subtraction angiogram immediately after completion of right hepatic artery RE with the catheter tip (“C”) in the right hepatic artery demonstrates significant vascular stasis and reflux of contrast into the left hepatic (“LHA”), proper hepatic, gastroduodenal (“GDA”), right gastroepiploic (“RGE”), common hepatic and splenic (“S”) arteries. **(C,D)** ^90^Y PET/CT depicts in high resolution, non-target activity in a non-random distribution conforming to the anatomy of the lower anterior gastric wall. **(E,F)** ^90^Y bremsstrahlung SPECT/CT shows concordant but subtle diffuse bremsstrahlung activity along the lower anterior gastric wall. The unexpected vascular stasis was attributed to reduced vascular capacitance due to the anti-angiogenic effects of recent sorafenib therapy.

**Figure 3:**
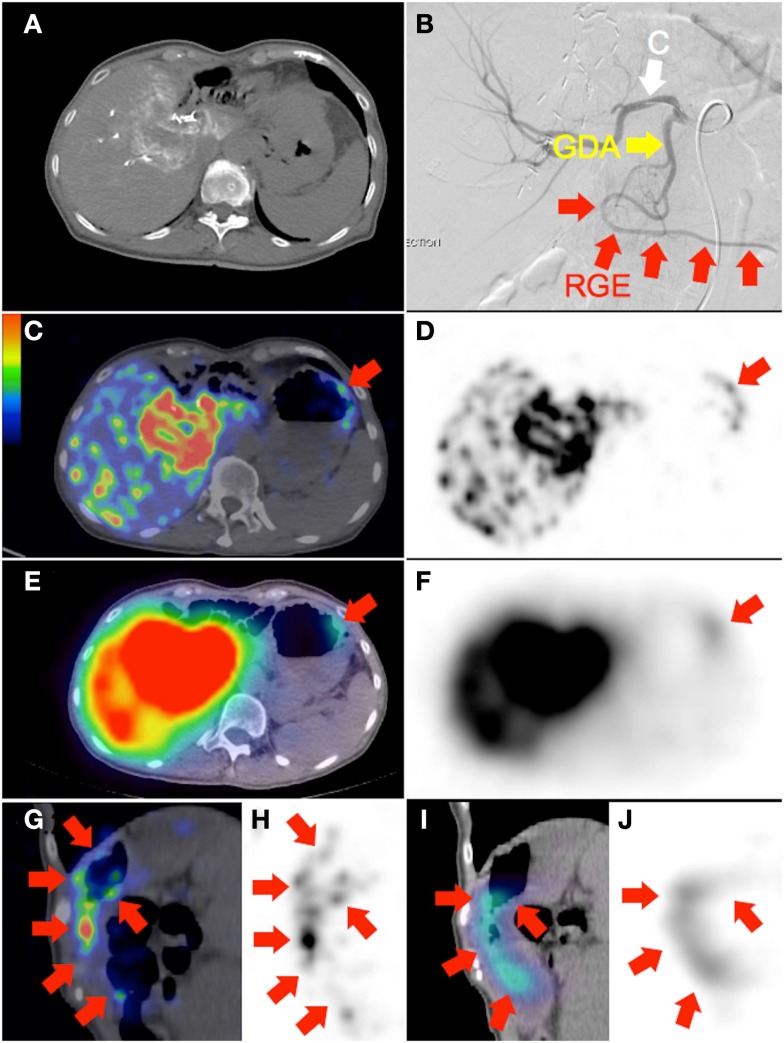
**(A)** Recurrent cholangiocarcinoma at the surgical margin of a previous left hemi-hepatectomy, seen on catheter-directed CT hepatic angiogram of the right hepatic artery. RE was performed with the catheter tip in the right hepatic artery, beyond the origin of the gastroduodenal artery. Prophylactic coil embolization of the gastroduodenal artery was not performed at the discretion of the interventional radiologist. **(B)** Post-RE digital subtraction angiogram with the catheter tip (“C”) position unchanged demonstrates significant vascular stasis and reflux of contrast in the common hepatic, gastroduodenal (“GDA”), and right gastroepiploic (“RGE”) arteries. **(C,D,G,H)** ^90^Y PET/CT in trans-axial and saggital planes depict in high resolution, non-target activity in a non-random distribution conforming to the anatomy of the gastric greater curve. **(E,F,I,J)** ^90^Y bremsstrahlung SPECT/CT shows concordant but less intense and diffuse non-target gastric activity.

**Figure 4:**
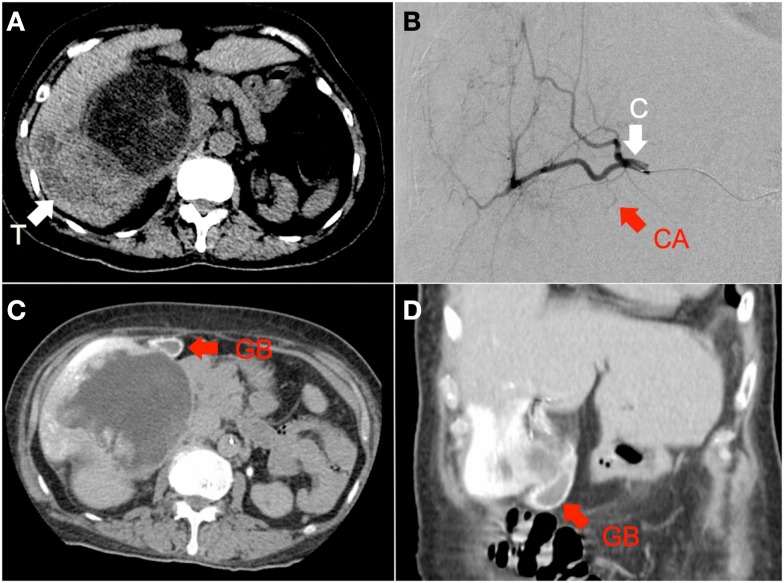
**(A)** Hepatocellular carcinoma of the right lobe (“T”) in a background of polycystic kidney and liver disease, seen on non-contrast-enhanced CT. **(B)** RE was performed with the catheter tip (“C”) in the right hepatic artery, proximal to the origin of the cystic artery (“CA”), seen here on digital subtraction angiography **(C,D)** Catheter-directed CT hepatic angiogram of the right hepatic artery demonstrates prominent contrast enhancement of the gallbladder wall (“GB”).

**Figure 5:**
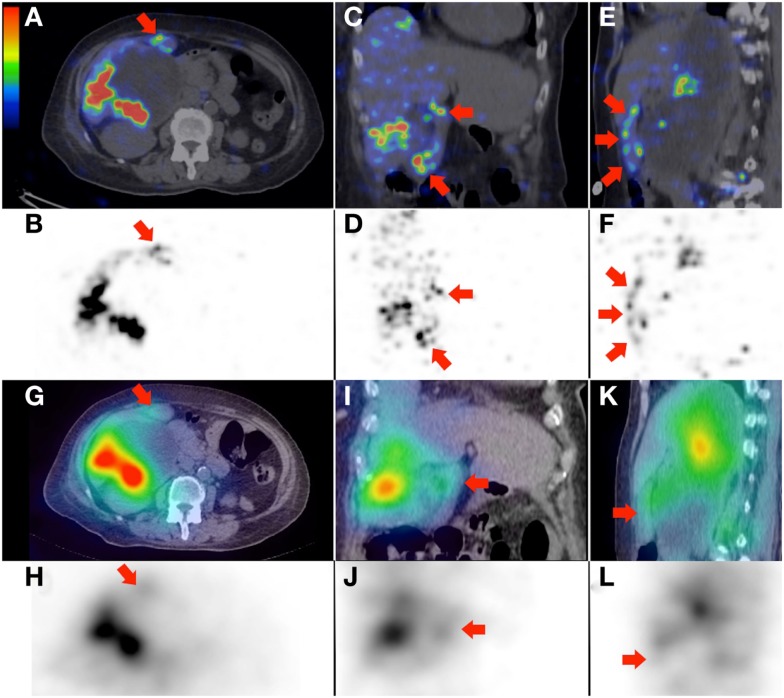
**(A–F)** ^90^Y PET/CT depicts in high resolution, non-target activity in a non-random distribution conforming to the anatomy of the gallbladder wall (arrows), shown here in trans-axial, coronal, and saggital planes, respectively. **(G–L)** ^90^Y bremsstrahlung SPECT/CT shows concordant but subtle diffuse non-target gallbladder activity.

**Figure 6:**
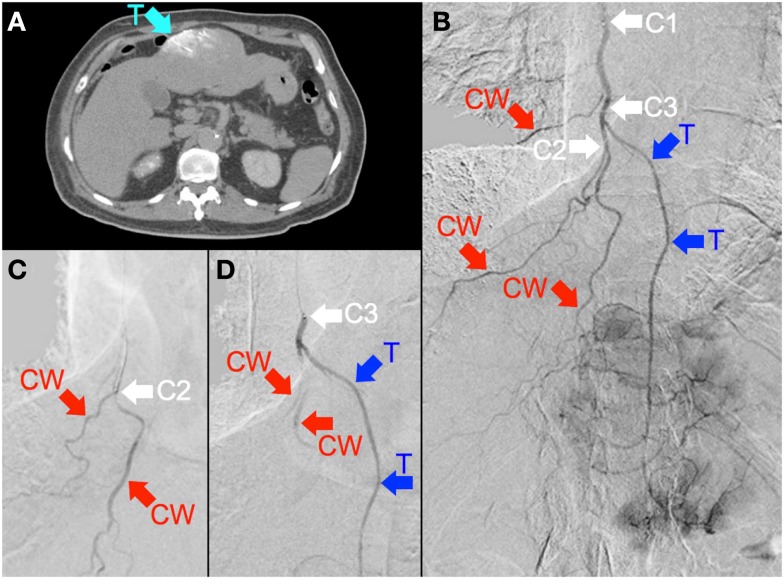
**(A)** Multifocal hepatocellular carcinoma with a large segment III tumor. Catheter-directed CT hepatic angiogram of the right internal mammary artery (RIMA) demonstrates blood supply to one-third of the large segment III tumor (“T”). **(B)** Digital subtraction angiogram (DSA) of the RIMA demonstrates the target arterial tree, with three major branches supplying the right lower anterior chest wall (“CW”) and a terminal branch supplying tumor (“T”). The tumor blush is seen distally. The catheter tip was positioned at the proximal RIMA (“C1”) for ^99m^Tc MAA injection. **(C)** To minimize non-target flow of ^90^Y resin microspheres to the chest wall, the catheter tip was advanced to the origin of two chest wall arteries (“C2”), where prophylactic bland embolization with gel foam slurry was performed until vascular stasis; coil embolization at this position was technically not possible due to vessel tortuosity. **(D)** The catheter tip was then pulled back slightly (“C3”), proximal to the origin of the tumor branch (“T”). RE was performed at this position with intermittent checks by DSA to ensure good forward flow and no reflux of contrast.

**Figure 7:**
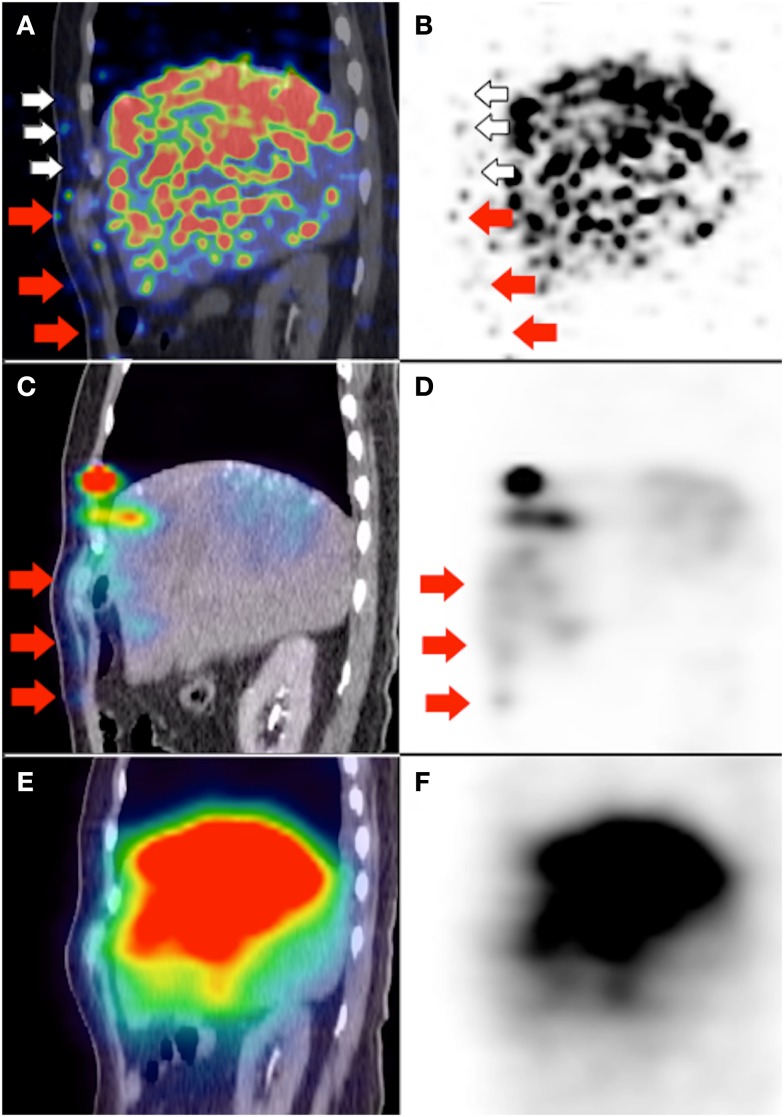
**(A,B)** ^90^Y PET/CT depicts in high resolution, non-target activity in a non-random distribution conforming to the anatomy of the right lower anterior chest wall (white and red arrows). **(C,D)** Pre-RE ^99m^Tc MAA SPECT/CT shows similar non-target activity along the right lower anterior chest wall (red arrows). The non-target chest wall activity on ^90^Y PET depicted by white arrows was not seen on ^99m^Tc MAA SPECT/CT due to altered arterial flow and biodistribution after prophylactic bland embolization of at-risk chest wall branches. **(E,F)** ^90^Y bremsstrahlung SPECT/CT was unable to detect any non-target activity along the right lower anterior chest wall due to its low image resolution.

**Figure 8:**
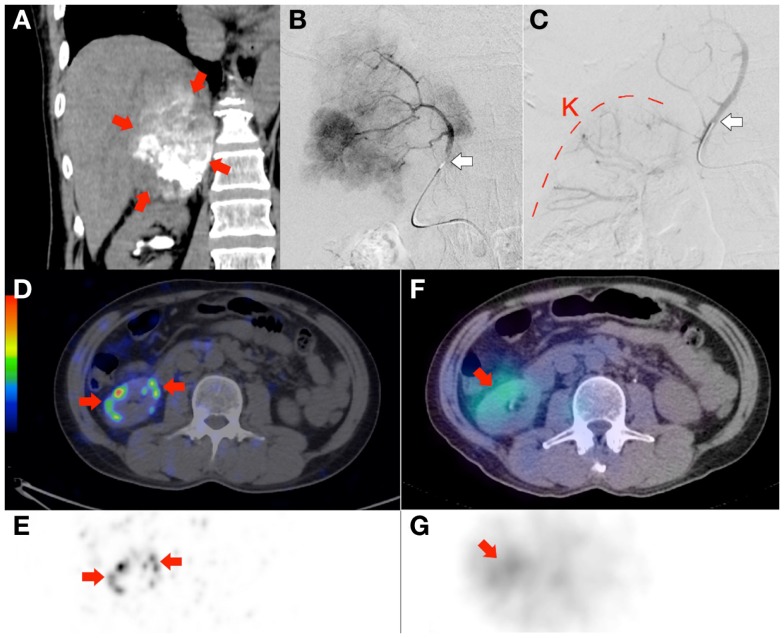
**Radioembolization to a site other than the liver**. Bulky right adrenal metastasis from chemorefractory gastrointestinal stromal tumor. **(A)** Catheter-directed CT angiography of the right inferior adrenal artery delineates the tumor (arrows) and the target arterial territory. **(B)** Digital subtraction angiogram (DSA) immediately prior to microsphere injection with the catheter tip (arrow) deep within the right inferior adrenal artery demonstrates good forward flow of contrast toward the tumor. **(C)** Post-RE DSA with no change to catheter tip (arrow) position demonstrates significant vascular stasis and reflux of contrast down the right inferior adrenal artery and distally into the terminal branches of the right renal artery. The renal cortex (curved dashed line) of the right kidney (“K”) can be seen. **(D,E)** ^90^Y PET/CT depicts in high resolution, non-target activity in a non-random distribution conforming to the anatomy of the right renal cortex (arrows). **(F,G)** ^90^Y bremsstrahlung SPECT/CT shows concordant but subtle diffuse non-target activity in the right renal cortex.

**Figure 9:**
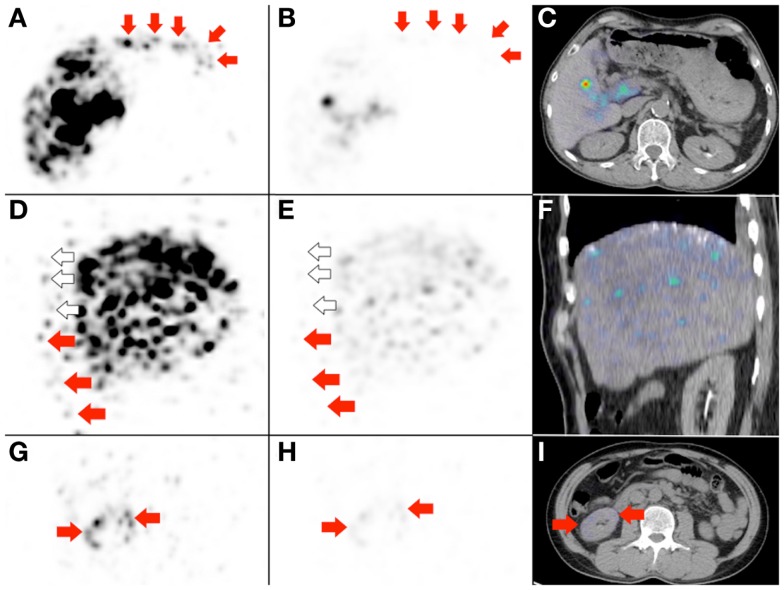
**Three examples to highlight the importance of deliberately increasing the background noise for non-target activity detection**. **(A,D,G)** These are duplicates of Figures 2D, 7B, and 8E, which show non-target activity in the lower anterior gastric wall, right lower anterior chest wall, and right renal cortex respectively, depicted with background noise deliberately increased. **(B,E,H)** If the PET visual display threshold had remained at the settings used for target activity assessment, the non-target activity will appear subtle on PET and undetectable on PET/CT **(C,F)**. **(I)** Right renal cortex non-target activity is barely detectable on PET/CT.

Figure 9: the importance of deliberately increasing the background noise for non-target activity detection.

## Concluding Remarks

With proper technique, the presence of background noise did not pose a problem for qualitative assessment of non-target activity by ^90^Y PET/CT. The image resolution of non-target activity by ^90^Y PET/CT was consistently superior to ^90^Y bremsstrahlung SPECT/CT in all cases.

## Author Contributions

Yung Hsiang Kao, Andrew E. H. Tan, David C. E. Ng, and Anthony S. W. Goh were involved in study design, implementation, analysis, and manuscript preparation. Richard H. G. Lo, Kiang Hiong Tay, and Bien Soo Tan were involved in radioembolization, angiographic analysis, and manuscript preparation. Pierce K. H. Chow was involved in clinical care and manuscript preparation.

## Conflict of Interest Statement

Yung Hsiang Kao, Kiang Hiong Tay, Pierce K. H. Chow, and Anthony S. W. Goh receive research funding from Sirtex Medical Singapore. Pierce K. H. Chow and Anthony S. W. Goh receive honoraria from Sirtex Medical Singapore. The publication fee for this article was covered by Sirtex Medical Australia.
